# Fibroblast Growth Factor Receptor, a Novel Receptor for Vegetative Insecticidal Protein Vip3Aa

**DOI:** 10.3390/toxins10120546

**Published:** 2018-12-18

**Authors:** Kun Jiang, Xiaoyue Hou, Lu Han, Tongtong Tan, Zhanglei Cao, Jun Cai

**Affiliations:** 1 Department of Microbiology, College of Life Sciences, Nankai University, Tianjin 300071, China; jiangkun@sdu.edu.cn (K.J.); xiaoyuehou@mail.nankai.edu.cn (X.H.); luhan0325@mail.nankai.edu.cn (L.H.); tantongtong@mail.nankai.edu.cn (T.T.); caozhanglei@mail.nankai.edu.cn (Z.C.); 2 State Key Laboratory of Microbial Technology, Shandong University, Qingdao 266237, China; 3 Key Laboratory of Molecular Microbiology and Technology, Ministry of Education, Tianjin 300071, China; 4 Tianjin Key Laboratory of Microbial Functional Genomics, Tianjin 300071, China

**Keywords:** Vip3Aa, Sf-FGFR, receptor

## Abstract

Vegetative insecticidal proteins (Vips), which are secreted by some *Bacillus thuringiensis* strains during vegetative growth, exhibit high virulence to many pests. Vip3A proteins have been used commercially both in some bio-insecticides and in transgenic crops; however, compared with insecticidal crystal proteins, the mechanism of action of Vip3A is still unclear. In this work, we indicated that the fibroblast growth factor receptor-like protein (Sf-FGFR) from the membrane of Sf9 cells could bind to Vip3Aa. The interaction between Vip3Aa and Sf-FGFR was confirmed by pull-down assays and dot blotting experiment in vitro. The binding affinity between Vip3Aa and extracellular regions of Sf-FGFR (GST-FGFR-N) was determined by microscale thermophoresis assay (MST). Moreover, Vip3Aa-Flag could be co-immunoprecipitated with Sf-FGFR-V5 ex vivo. Furthermore, knockdown of *Sf-FGFR* gene in Sf9 cells resulted in reducing the mortality of those cells to Vip3Aa. In summary, our data indicated that Sf-FGFR is a novel receptor for Vip3Aa.

## 1. Introduction

*Bacillus thuringiensis* (Bt) is a gram-positive, soil-dwelling bacterium that naturally produces insecticidal crystal proteins (ICPs) during sporulation. The ICPs are highly specific against a variety of insects, as well as nematodes, mites, protozoa, and some human-cancer cells [1,2]. They have been used worldwide by traditional spray approaches or transgenic crops [3,4]. The mechanism of action of ICPs has been studied extensively, and several types of membrane proteins have been identified as receptors for ICPs, such as aminopeptidase N (APN), the cadherin-like proteins, alkaline phosphatases, and ABC transporter [5,6]. So far, the pore-forming model is the widely accepted mode of action for ICPs. The action of ICPs is a multistep process. After ingestion by a susceptible larva, the environment of the midgut promotes crystal solubilization, protoxin release, and toxin production. After that, toxins bind to the specific receptors on the brush border membrane of midgut cell that facilitate the formation of an oligomeric structure, followed by pores formed in the cell membrane, resulting in the death of the insect [2]. With the extensive applications of ICPs, however, the cases of insect resistance to them are constantly emerging [2,3,4].

The vegetative insecticidal proteins (Vips), which were first found by Estruch et al. in 1996, were produced by certain Bt strains at the vegetative stage, and are considered novel insecticidal toxins because of their genetically distinct from known ICPs [7,8]. Studies have shown that Vip3A and ICPs recognize different receptors, indicating that their combination will not only diversify the range of target pests but also decrease the chances of cross-resistance [8,9]. Nowadays, the co-expression of Vip3A proteins with ICPs has been used commercially, both in traditional Bt-based insecticides and in transgenic crops [8,10,11]. However, compared with ICPs, the mechanism of action of Vip3A is still unclear. In particular, limited studies are currently available to address the specific receptors of Vip3A.

Singh et al. found that the ribosomal protein S2 in Sf21 cells can function as an interacting partner protein of Vip3A [12]. In our recent work, we confirmed that the scavenger receptor class C like protein (Sf-SR-C) is a specific receptor for Vip3Aa in Sf9 cells [9]. In addition, we also identified 36 other proteins besides Sf-SR-C from the extracted Sf9 cell membrane proteins, which could bind to Vip3Aa. The fibroblast growth factor receptor-like protein (Sf-FGFR) is one of the 36 proteins, which suggests that it may be a potential receptor of Vip3A. In this study, the specificity of the interaction between Sf-FGFR and Vip3A has been examined by a combination of in vitro and ex vivo assays. Our results confirmed that Sf-FGFR from Sf9 cells is a novel receptor for Vip3Aa.

## 2. Results

### 2.1. Interacting Partners to Vip3Aa Include Sf-FGFR

**Figure 1:**
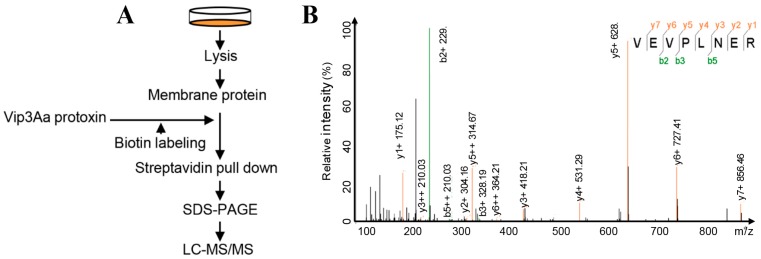
Sf-FGFR is presumed to interact with Vip3Aa. (**A**) A schematic diagram of the overall workflow to identify Vip3Aa interacting proteins from the extracts of Sf9 cell membrane proteins. Experimental details are described in the Method section. (**B**) The MS/MS spectrum of the Sf-FGFR peptide identified.

In our previous study, to identify the interacting partners of Vip3Aa, we used the affinity magnetic bead method, coupled with high-performance liquid chromatography-tandem mass spectrometry (HPLC-MS/MS) [9]. The Vip3Aa protein was used as a bait; we identified the fibroblast growth factor receptor-like protein (Sf-FGFR) from membrane-proteins in Sf9 cells that could bind to Vip3Aa (Figure S1). In this work, we extracted membrane-proteins of Sf9 cells and re-examined the interacting partners to Vip3Aa in the same way to avoid contingency (Figure 1A). Results (Table S1) of protein sequence database searching indicated that 35 proteins could bind to Vip3Aa, which also contained Sf-FGFR (Figure 1B). However, most of Vip3A binding proteins identified from the extracted Sf9 cell membrane are ribosomal proteins or cytoplasmic proteins, which were not likely to be a receptor of Vip3A. Sf-FGFR is another real membrane protein except for Sf-SR-C; therefore, we speculated that Sf-FGFR protein may be a novel receptor of Vip3Aa. 

### 2.2. The Extracellular Regions of Sf-FGFR Binds to Vip3Aa

**Figure 2:**
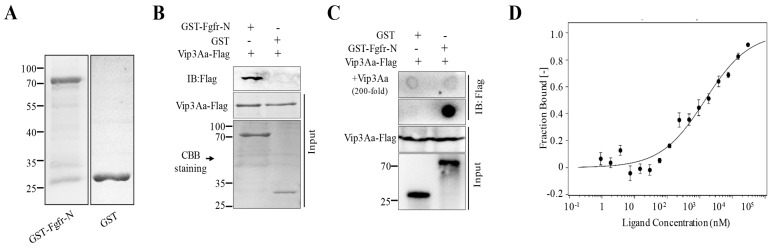
The extracellular regions of Sf-FGFR could bind to Vip3Aa. (**A**) The purified GST-FGFR-N and GST protein. (**B**) The purified GST-FGFR-N and GST were mixed with Vip3Aa-Flag, respectively, and then the GST-Sepharose affinity beads were added followed by immunoblotting (IB) with the primary antibody (Mouse anti-Flag). (**C**) GST and GST-FGFR-N proteins were dotted on a PVDF membrane, respectively, and were incubated with Vip3Aa-flag (100 nM) or Vip3Aa-flag plus excess unlabeled Vip3Aa (200-fold), followed by immunoblotting with the primary antibody (Mouse anti-Flag). (**D**) The binding affinity of Vip3Aa with GST-FGFR-N was analyzed with MST. The labeled Vip3Aa was kept constant at 10 nM and the GST-FGFR-N was titrated from 0.3 nM to 10 µM. The equilibrium dissociation constant (Kd, mean ± SD) was the fitting result of three independent experiments.

To ascertain the role of the Sf-FGFR protein that can bind to Vip3Aa, we cloned the Sf-FGFR gene (GenBank accession no. KX979914) from the cDNA of Sf9 cells. However, we failed to express full-length Sf-FGFR in *E. coil* BL21 (DE3) cells due to the fact that the cells almost stopped growing in LB containing IPTG. The FGFR family members have three important domains that are similar to most receptor tyrosine kinases, including an intracellular tyrosine kinase domain, a single transmembrane domain, and an extracellular ligand-binding domain [13] (Figure S2). The extracellular part is composed of two or three immunoglobulin-like domains, which constitute the ligand-binding site [14]. Thus, we purified the extracellular regions of Sf-FGFR (Sf-FGFR-N, aa 27–415) with a glutathione-S-transferase (GST) tag and the protein GST-FGFR-N was obtained (Figure 2A) to test whether Vip3Aa could interact with Sf-FGFR-N. 

Pull-down assay demonstrated that Vip3Aa-Flag could bind to GST-FGFR-N, while GST could not (Figure 2B). Further studies of the specific binding between GST-FGFR-N and Vip3Aa-Flag were performed using dot blotting assay. Results of dot blotting were shown in Figure 2C. Vip3Aa-Flag could combine with GST-FGFR-N, and the specificity of binding between Vip3Aa and GST-FGFR-N was further confirmed by excess Vip3Aa (200-fold, without Flag tag) protein competitive assay. Furthermore, to determine the binding affinity between GST-FGFR-N and Vip3Aa, microscale thermophoresis assay (MST) [15] was performed, and the estimated dissociation constant (Kd) was 1.43 ± 0.87 μM (Figure 2D). 

### 2.3. Ex vivo Binding Study of Sf-FGFR and Vip3Aa

**Figure 3:**
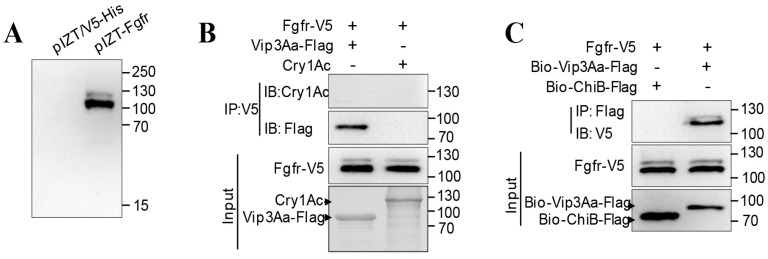
Sf-FGFR can interact with Vip3Aa. (**A**) The recombinant plasmid pIZT-FGFR and the vector pIZT/V5-His was transiently transfected into Sf9 cells, respectively. Cells were collected for immunoblotting with the primary antibody (rabbit anti-V5) at 48 h. (**B**) The lysate of Sf9 cells transfected with pIZT-FGFR was incubated with Cry1Ac or Vip3Aa-Flag, Sf-FGFR was immunoprecipitated (IP) with the primary antibody (rabbit anti-V5), and the primary antibody used to detect Cry1Ac and Vip3Aa-Flag was an anti-Cry1Ac antibody and anti-Flag antibody respectively. (**C**) Biotin-labeled Vip3Aa-Flag (Bio-Vip3Aa-Flag) or Biotin-labeled ChiB-Flag (Bio-ChiB-Flag) was incubated with the lysate of Sf9 cells transfected with pIZT-FGFR, immunoprecipitated with Streptavidin Mag Sepharose. Sf-FGFR protein was detected by immunoblotting with the primary antibody (rabbit anti-V5).

Further studies of the interaction between full-length Sf-FGFR and Vip3Aa were performed using ex vivo binding studies. *Sf-FGFR* was inserted into plasmid pIZT/V5-His, creating recombinant plasmid pIZT-FGFR (Figure 3A). After pIZT-FGFR was transiently transfected into Sf9 cells, the Sf-FGFR protein with a V5 tag was expressed. It is evident from immunoprecipitation analysis with the primary antibody (rabbit anti-V5) that Vip3Aa-Flag could be co-immunoprecipitated with Sf-FGFR-V5 (Figure 3B), while Cry1Ac did not. Moreover, by way of affinity, magnetic bead method and immunoblotting, biotin-labeled Vip3Aa-Flag could interact with Sf-FGFR-V5, while control biotin labeled ChiB-flag (Chitinase B secreted by Bt) did not (Figure 3C). Taken together, these results showed Vip3Aa can bind to Sf- FGFR ex vivo.

### 2.4. Vip3Aa-RFP could Co-localize with Sf-FGFR on the Suface of Sf9 Cells

**Figure 4:**
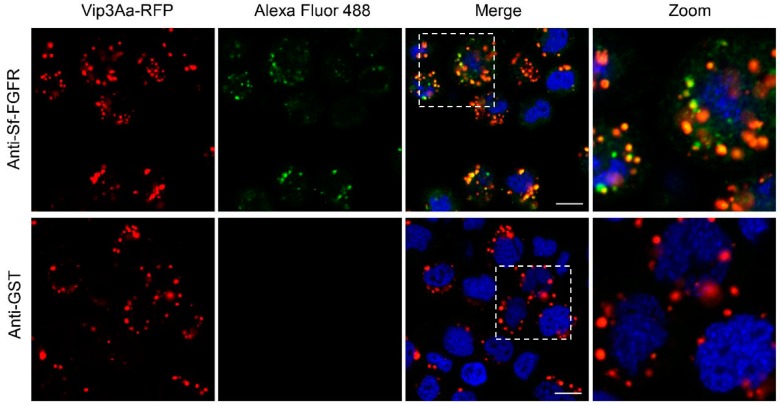
Vip3Aa could co-locate with Sf-FGFR. Confocal images showing the localization of Sf-FGFR (green) and Vip3Aa-RFP (red) on the surface of Sf9 cells or in the cells. The anti-Sf-FGFR-N polyclonal antibody and Alexa Fluor 488-conjugated anti-rabbit antibody were used to show the location of Sf-FGFR in Sf9 cells. Anti-GST polyclonal antibodies were used as the control. Nuclei were stained with DAPI (blue). Scale bar, 10 μm.

We also carried out co-localization assays to detect the interaction between Vip3Aa and Sf-FGFR. The Sf9 cells were treated with Vip3Aa-RFP (a fusion protein of Vip3Aa protoxin and red fluorescence protein) for 6 h, and then the distribution of Vip3Aa and Sf-FGFR was monitored by immunofluorescent staining. As shown in Figure 4, most of the dots of Vip3Aa-RFP could co-locate with Sf-FGFR on the cell membrane or in the cells, especially in the dots that were Sf-FGFR-rich. In the control experiment that the anti-GST polyclonal antibody was used, we did not observe similar phenomena, which further suggested that Vip3Aa could interact with Sf-FGFR.

### 2.5. Reducing the Expression of Sf-FGFR Gene in Sf9 Cells Decreases the Cell Sensitivity to Vip3Aa

**Figure 5:**
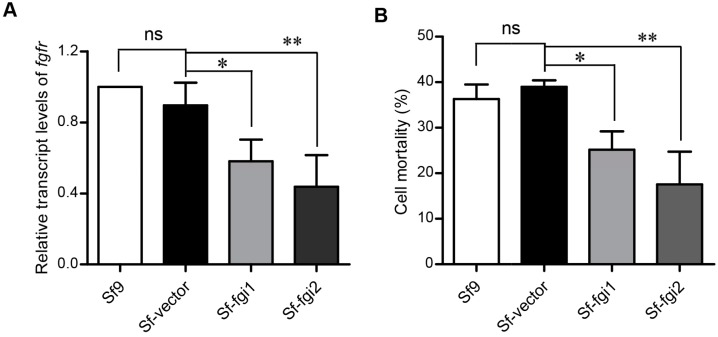
Reducing the expression of Sf-FGFR gene in Sf9 cells decreased the sensitivity of these cells to Vip3A. (**A**) The relative transcript levels of FGFR genes in the Sf-pIZT, Sf-Fgi1 and Sf-Fgi2 cells determined by qRT-PCR analysis. (**B**) Cell mortality of Sf9, Sf-pIZT, Sf-Fgi1 and Sf-Fgi2 cells after treated with Vip3Aa (50 µg/mL) for 48 h. The results are presented as the means ± SD of values obtained in three independent experiments. Statistical significance was calculated using the ANOVA and Tukey’s test; ns, non-significant; * *P* < 0.05, ** *P* < 0.01. ns: non-significant.

To validate the role of Sf-FGFR in the potency of Vip3Aa to Sf9 cells, we tried to knock down the expression of endogenous *Sf-FGFR* gene in Sf9 cells [16]. Two plasmids, pIZT-Fgi1 and pIZT-Fgi2 (Figure S3), which can generate fragments of double-stranded RNA (dsRNA) of *Sf-FGFR* gene, were stably transfected into Sf9 cells, creating cell lines Sf-Fgi1 and Sf-Fgi2, respectively. The quantitative real-time reverse transcription PCR (qRT-PCR) [17] was conducted to analyze the transcription levels of *Sf-FGFR* gene, and the *actin* gene acted as the endogenous control. As shown in Figure 5A, the mRNA levels of *Sf-FGFR* gene in the Sf-Fgi1 and Sf-Fgi2 cells were 64.2% and 54.3% of the cells, which were stably transfected with pIZT/V5-His (Sf-pIZT cells), respectively. The results of the CCK-8 cytotoxicity assay [9] showed that the reduction in the expression of *Sf-FGFR* gene correlated well with the observed reduced toxicity of Vip3Aa in the Sf-Fgi1 and Sf-Fgi2 cells (Figure 5B). It suggested that reducing the expression of the *Sf-FGFR* gene in Sf9 cells decreased cell sensitivity to Vip3Aa.

## 3. Discussion

Vip toxins, which are generally regarded safe based on their specificity toward the target pest and the lack of significant effects on non-target organisms, are regarded as second-generation insecticidal proteins for their insecticidal mechanism different from ICPs [8,9]. The combined application of Vip3A and ICPs is a suitable pyramid strategy to control pests, as well as to reduce the chances of cross-resistance. Vip3A accounts for the largest number of Vip proteins and has been applied in practice [8]. However, the insecticidal mechanisms of Vip3A are still uncertain. In this study, pull-down, dot blotting, and co-immunoprecipitation assays were performed to detect the interaction of Vip3Aa and Sf-FGFR and their binding affinity was determined by MST assay. In addition, the expression of the *Sf-FGFR* gene reduced in Sf9 cells leads to decrease in cell sensitivity to Vip3Aa. As a result, we confirmed that Sf-FGFR is a novel receptor for Vip3Aa.

The fibroblast growth factor receptors (FGFRs) belong to a subfamily of receptor tyrosine kinases and are mainly studied in mammalian cells [14]. They mainly comprise of four family members—FGFR1, FGFR2, FGFR3, and FGFR4—which play essential roles, such as cell proliferation, survival, migration, and differentiation, in many aspects of cellular physiology [18]. Once in interaction with fibroblast growth factors (FGFs), they can initiate downstream signal transduction, such as activation of PLCγ, MAPK, AKT, and STAT cascade [19]. Through sequence alignment, we found that Sf-FGFR is most similar to FGFR1. It has been reported that cellular apoptosis was obviously observed after FGFR1-amplified lung cancer cell lines were treated with the specific inhibitor (PD173074) [20]. Pardo et al. have demonstrated that oral administration of the FGFR inhibitor suppressed tumor growth and increased apoptosis in SCLC xenograft mouse models [21]. Treatment of FGFR1OP2–FGFR1-positive cells with inhibitors against FGFR1 also led to apoptosis [22]. 

Studies have shown that Vip3Aa can trigger apoptosis in sensitive cells [23,24]. Therefore, whether the binding of Vip3Aa to Sf-FGFR affects the relevant signaling pathways to induce apoptosis needs further study.

Moreover, by immunofluorescence co-localization experiment, we found that Vip3Aa and Sf-FGFR could co-localize on the surface of Sf9 cells as well as inside of Sf9 cells, indicating that Vip3Aa and Sf-FGFR could internalize into Sf9 cells together. It has been reported that one of the modulations of the FGFR signaling is that the activated FGF–FGFR complex is terminated by internalization and degradation in lysosomes [25]. Our recent works have demonstrated that the Sf-SR-C could mediate Vip3Aa into Sf9 cells via endocytosis and the internalization of Vip3Aa correlates with its insecticidal activity [9]. Thus, whether Sf-FGFR can directly mediate Vip3Aa into Sf9 cells and whether their internalization is related to the toxicity of Vip3Aa or the protection strategy of cells need further study.

Furthermore, we have certified that the Sf-SR-C is a specific receptor for Vip3Aa. Here, we further proved that Sf-FGFR is a novel receptor of Vip3Aa. More research is needed to test whether Sf-SR-C and Sf-FGFR are related in the process of Vip3Aa acting on Sf9 cells. In addition, comparing the results of mass spectrometry (Table S1) with our previous results [9] (Table S1), we found that in addition to Sf-SR-C, Sf-FGFR and the ribosomal proteins, nine other proteins were simultaneously identified (Table S2). Whether there are other receptors of Vip3Aa among them also need to be studied.

## 4. Conclusions

In summary, via in vitro, ex vivo and cytotoxicity assay, we confirmed that Sf-FGFR is a specific receptor for Vip3Aa. Our results not only provide new research contents for the molecular mechanism of Vip3Aa, but also promote the clarity of the insecticidal mechanism of Vip3Aa.

## 5. Materials and Methods

### 5.1. Bacterial Strains and Cell Lines 

*Escherichia coli* strains DH5α and BL21 (DE3), which were cultured at 37℃ in LB (lysogeny broth), were used for plasmid constructions and protein purification, respectively. The *Spodoptera frugiperda* Sf9 cells were cultured in SFX-Insect cell culture medium (HyClone, Logan, UT, USA) supplemented with 10% fetal bovine serum (FBS) (GIBCO, Grand Island, NY, USA), at 28℃. Sf9 cells were changed medium every 3 days and sub-cultured after 80–90% confluence.

### 5.2. Chemicals

RIPA buffer (#9806S), mouse anti-Flag (#8146), rabbit anti-V5 (#13202), goat anti-rabbit IgG-HRP conjugate (#7074), and Alexa Fluor 488 goat anti-rabbit IgG (#4412) were obtained from Cell Signaling Technology (Beverly, Boston, MA, USA). Goat anti-mouse IgG-HRP conjugate (#sc-2005), rabbit polyclonal anti-GST (#bs-2735R), Anti-V5-Dylight 488 conjugate (#MA5-15253-D488) was purchased from Santa Cruz (Santa Cruz, TX, USA), Bioss (Boston, MA, USA), and Invitrogen (Carlsbad, CA, USA), respectively. Anti-Sf-FGFR-N polyclonal antibodies were generated by immunizing rabbits with purified GST-FGFR-N.

### 5.3. Protein Purification

The *Sf-FGFR-N* gene fragment, which was amplified using primer FGFR-N-F and FGFR-N-R, was inserted into the pGEX-6P-1 vector using a pEASY^®^-Uni Seamless Cloning and Assembly Kit (TransGen, Beijing, China) after double digestion of the vector with *Bam*HI and *Xho*I, resulting in a glutathione-S-transferase (GST) fusion. The expression of Vip3Aa and Vip3Aa-RFP was performed using the previously described method [9].

The recombined plasmids were transformed into *E. coli* BL21 (DE3) and corresponding transformants were used for protein purification. GST-tagged protein was purified by using GST-Sepharose affinity column (GE Healthcare, Fairfield, CT, USA). The purified protein was dialyzed at 4 ℃ against a buffer containing 25 mM Tris-HCl (pH 8.0), 150 mM NaCl and 5% glycerol with several buffer changes.

All the primers and plasmids used in this study are listed in Table 1 and Table 2, respectively.

### 5.4. Microscale Thermophoresis (MST) Assay

The binding affinity between GST-FGFR-N and Vip3Aa was determined by MST assay [9]. The purified GST-FGFR-N and Vip3Aa proteins were dialyzed against 25 mM HEPES (pH 7.5), 0.05 (*v*/*v*) % Tween-20, and 150 mM NaCl. Vip3Aa was labeled with the Monolith NT™ Protein Labeling Kit (#L008, NanoTemper, Munich, Germany) according to the protocol. Labeled Vip3Aa (10 nM) was hatched with 0.3 nM to 10 µM GST-FGFR-N protein for 20 min at room temperature, respectively. Then, samples were loaded into capillaries and analyzed via a NanoTemper^®^ Monolith NT.115 Pico (NanoTemper Technologies GmbH) at 25℃. Furthermore, the LED power and the laser power were set to 60% and 10%, respectively. The software MO Affinity Analysis v2.2.2 (NanoTemper) was used to normalize the fluorescence signal and fit the Hill equation. The whole procedure was performed three times to produce independent triplicates for each sample.

### 5.5. Plasmid Construction, Preparation, and Transfection

In order to silence *Sf-FGFR* gene, the plasmids were constructed as described previously [14]. Briefly, fragments of the *Sf-FGFR* gene (nucleotides [nt] 60 to 679, dsRNA1s) and 500 bp from the *Sf-FGFR* gene complementary strand (nt 559 to 60, dsRNA1as) were amplified using the primers Fgi1-Up-F and Fgi1-Up-R (for dsRNA1s) or Fgi1-Do-F and Fgi1-Do-R (for dsRNA1as). The dsRNA1s and dsRNA1as were inserted into the pIZT/V5-His vector in tandem using a pEASY^®^-Uni Seamless Cloning and Assembly Kit after *Kpn*I–*Age*I double digesting the vector (pIZT-Fgi1). The pIZT-Fgi2 was constructed as pIZT-Fgi1 by using the primer sets Fgi2-Up-F and Fgi2-Up-R or Fgi2-Do-F and Fgi2-Do-R. We generated stable *Sf-FGFR* gene silencing Sf9 cells by transfection with pIZT-Fgi1 or pIZT-Fgi2 using the Cellfectin II reagent and PLUS™ Reagent. Invitrogen Zeocin (500 μg/mL) was added into the culture medium 48 h post- transfection. The culture medium was replaced every four days. Three weeks after Invitrogen Zeocin selection, the expression level of *Sf-FGFR* gene was analyzed by qRT-PCR analysis. 

### 5.6. Mass Spectrometry

ProteoExtract Transmembrane Protein Extraction Kit (Novagen, Madison, WI, USA) was used to extract the membrane proteins of Sf9 cells. Vip3Aa was labeled with biotin as described previously [9]. Fifty microliters of Streptavidin Mag Sepharose beads (GE Healthcare, Fairfield, CT, USA) were washed with PBS and incubated with 0.2 mg Vip3Aa (biotin-labeled, Bio-Vip3Aa) for 1 h at 4 °C. Then, the Vip3Aa tagged beads were mixed with 0.5 mL of membrane-proteins of Sf9 cells for 3 h at 4 °C. After several washed with PBS buffer, the precipitants were boiled with SDS loading buffer and then analyzed by SDS-PAGE. The corresponding band represented for Vip3Aa was removed away and the remaining bands were sent for LC-MS/MS (tandem mass spectroscopy) analysis.

### 5.7. Western Blotting and Immunoprecipitation

Cells were collected and lysed in 0.5 ml RIPA buffer. After centrifugation at 14 000× *g* for 20 min, the lysate (30 μL) was co-incubated with Vip3Aa-Flag (10 μg) at 4°C for 2–3 h. The sample was immunoprecipitated with 5 µL rabbit anti-V5 overnight at 4 °C, and then 40 µL of protein G agarose beads (Santa Cruz, Texas, USA) were added for an additional 4 h. Subsequently, the immune complexes were washed five times with PBS buffer containing 2 mM Na_2_HPO_4_, 0.5 mM KH_2_PO_4_, 1.3 mM KCl, 135 mM NaCl, pH 7.4 and boiled with loading buffer for 10 min and separated on a 12% polyacrylamide gel and transferred to a PVDF membrane (Millipore, Milan, Italy). Mouse anti-flag (primary antibody) and Goat anti-mouse IgG-HRP conjugate (HRP-coupled secondary antibody) were applied to detect the corresponding protein. The PVDF membrane was visualized with Immobilon Western Chemiluminescent HRP Substrate (Millipore, Milan, Italy).

### 5.8. Dot Blotting and Pull-down Assay

Five microliters of GST-FGFR-N and GST (0.1 nmol) were separately dotted onto a PVDF membrane. Afterward, the membrane was blocked with 5% skimmed milk and incubated in Vip3Aa-flag (100 nM) for 1.5 h at room temperature. After three washes in PBS buffer containing 0.1% Tween-20 (TBST), 200-fold excess Vip3Aa was used in competition binding experiments. The subsequent steps are the same as the protocol of western blotting.

The GST-Sepharose affinity beads were incubated with the GST-FGFR-N solution (0.4 nmol) at 4°C for 3 h and then washed three times with PBS to remove unbound protein. After incubated with Vip3Aa-flag (100 nM), the beads were washed five times in PBS. The precipitated components were boiled with 5 × SDS loading buffer for 10 min and detected by western blotting.

### 5.9. Immunostaining and Confocal Microscopy

Sf9 cells were grown to 70% confluence in Laser confocal culture dishes. After treatment with Vip3Aa-RFP, the cells were washed three times with PBS to remove unbound proteins and fixed with 4% paraformaldehyde at 37 °C for 30 min. After washing three times with PBS, the cells were then permeabilized (0.5% Triton X-100) at room temperature for 30 min and immunostained. The anti-Sf-FGFR-N polyclonal antibody (primary antibodies) and Alexa Fluor 488-conjugated anti-rabbit antibody (secondary antibody) were diluted in 5% bovine serum albumin. Nuclei were labeled for 30 min with DAPI (Sigma, St. Louis, MO, USA). Cell images were captured using a Zeiss.LSM710 confocal microscope.

### 5.10. Cytotoxicity Assays

Cell viability was detected using the CCK-8 Counting Kit (Dojindo, Kumamoto, Japan). Cells in good condition were selected and incubated for 24 h at 28 °C in the 96-well plates with 100 μL cell suspensions (1 × 10^5^ cells) in each hole. Vip3Aa solution (50 μg/mL) or sterile dialysis buffer was added to each well for 48 h at 28 °C. Then, 10 μL WST-8 reagent was added to each well and the plates were incubated in the darkness for 2 h at 28 °C. The absorbance was measured at 450 nm using a microplate reader (PerkinElmer, Boston, MA, USA). The experiments were performed in sextuplicate and were repeated at least three times. Cell viability (%) = absorbance of Vip3Aa treated group/absorbance of sterile dialysis buffer treated group × 100%.

## Figures and Tables

**Table 1:** Primers used in this study.

Primer Name	Sequence (5′→3′)	Function
pIZT-Fgfr-F pIZT-Fgfr-R Fgi1-Up-F Fgi1-Up-R Fgi1-Do-F Fgi1-Do-R Fgi2-Up-F Fgi2-Up-R Fgi2-Do-F Fgi2-Do-R Fgfr-N-F Fgfr-N-R Sf-actin-RT-F Sf-actin-RT-R Sf-Fg-RT-F Sf-Fg-RT-R	TCGAATTTAAAGCTTGGTACAATGGTAATGAGTCTCGCCGCCATCGC AGGCTTACCTTCGAACCGCGGCTTGATGAAGGGGAAGTCACTA CGAATTTAAAGCTTGGTACGGCAACGGGGTGTCTCGCTCAAAC ATGAGAAACAAGATTACCAAGTTATGTTCGGCGTAGGGTT GAACATAACTTGGTAATCTTGTTTCTCATCTATATGACC AATGGTGATGGTGATGATGAGGCAACGGGGTGTCTCGCTCAAACC CGAATTTAAAGCTTGGTACAAGGTGCTCGGAGAAGGAGAGTTTG AGGATGTCCACAGAGGAGCACGCCGAACGACCAGACAT TTCGGCGTGCTCCTCTGTGGACATCCTTGGCCAGACCGA AATGGTGATGGTGATGATGAAAGGTGCTCGGAGAAGGAGAGTTTG CTGTTCCAGGGGCCCCTGGGACAAACCAGAGAAATTGTCTTGG TCAGTCACGATGCGGCCGCTCCTATGTGTGCTTTCCATGGTCTGGCG TCCTCCGTCTGGACTTGGC CTTCTCCTTGATGTCACGAACG GGCTGTGATAGTGACGCATTG CTTCGCCCGTAGCAGTAGG	Fgfr cloning Fgfr cloning Fgfr RNAi Fgfr RNAi Fgfr RNAi Fgfr RNAi Fgfr RNAi Fgfr RNAi Fgfr RNAi Fgfr RNAi Fgfr-N cloning Fgfr-N cloning Actin qRT-PCR Actin qRT-PCR Fgfr qRT-PCR Fgfr qRT-PCR

**Table 2:** Plasmids used in this study.

Plasmids	Relevant Characteristics	Reference
pET-Vip	*Vip3Aa* gene cloned into pET-28a (+), His tag binding C-terminal of Vip3Aa	[9]
pET-Vip-flag	*Vip3Aa* gene cloned into pET-28a (+), Flag-His tag binding C-terminal of Vip3Aa	[9]
pET-ChiB-flag	*ChiB* gene cloned into pET-28a (+), Flag-His tag binding C-terminal of ChiB	[9]
pET-Vip-RFP	*RFP* gene cloned into pET-Vip, RFP binding C-terminal of Vip3Aa	[9]
pIZT/V5-His	Expression vector, Zeocin^r^, C-terminal V5-His tag	Invitrogen
pIZT-Fgfr	*Sf-Fgfr* gene cloned into pIZT/V5-His, V5-His tag binding C-terminal of Sf-Fgfr	This study
pIZT-fgi1	Fragment of *Sf-Fgfr* gene (60-679) and the reverse complemented fragment of *Sf-Fgfr* (559-60) cloned into pIZT/V5-His	This study
pIZT-fgi2	Fragment of *Sf-Fgfr* gene(1600-2219) and the reverse complemented Fragment of *Sf-Fgfr* (2099-1600) cloned into pIZT/V5-His	This study
pGEX-6P-1	Expression vector, Amp^r^, N-terminal GST tag	Lab collection
pGEX-Fgfr-N	Extracellular sequence of *Sf-Fgfr* gene (Fgfr-N) cloned into pGEX-6P-1, GST tag binding N-terminal of Fgfr-N	This study

Ampr: ampicillin resistance, Kanr: kanamycin resistance, Zeocinr: zeocin resistance.

## Supplementary Materials

The following are available online at http://www.mdpi.com/2072-6651/10/12/546/s1, Table S1: Proteins identified from protein sequence database based on MS/MS results, Table S2: The proteins identified in both two-mass spectrometry except Sf-SR-C, Sf-FGFR, and the ribosomal proteins, Figure S1: Peptides of Sf-FGFR identified in our previous mass spectrometry: DGVLVDSQR and KTLTQK, Figure S2: Schematic representation of Sf-FGFR, indicating different domains. Immunoglobulin-like domain I (IgI), IgII, IgIII, and the tyrosine kinase domain. Sig-seq: signal peptide sequence, TM: transmembrane. The numbers represent the number of amino acids at the position. The domains of Sf-FGFR were predicted by protein Blast in NCBI and the Sig-seq and TM regions were predicted by the software of TMHMM 2.0 (http://www.cbs.dtu.dk/services/TMHMM-2.0/), Figure S3: Schematic diagram of dsRNA expression plasmids: pIZT-Fgi1 and pIZT-Fgi2.
