# Supplementary figures and images for: Fibroblast Growth Factor Receptor, a Novel Receptor for Vegetative Insecticidal Protein Vip3Aa
Source: Toxins (Basel). 2018 Dec 18;10(12):546. doi: 10.3390/toxins10120546 (PMC6315849; doi:10.3390/toxins10120546)

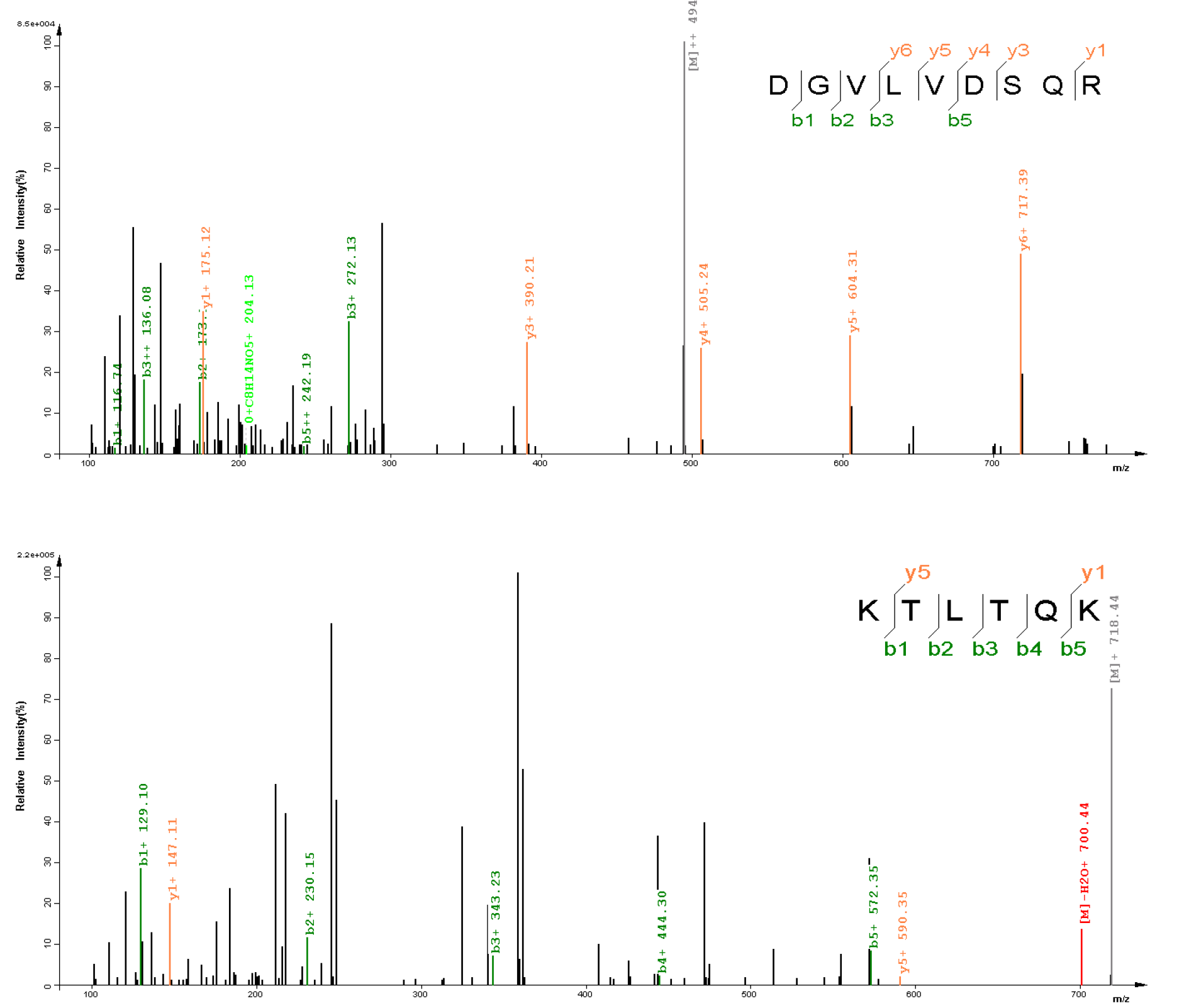

Supplement: Supplementary file 1 [file toxins-10-00546-s001.zip › Supplementary Materials/Figture S1.tif]

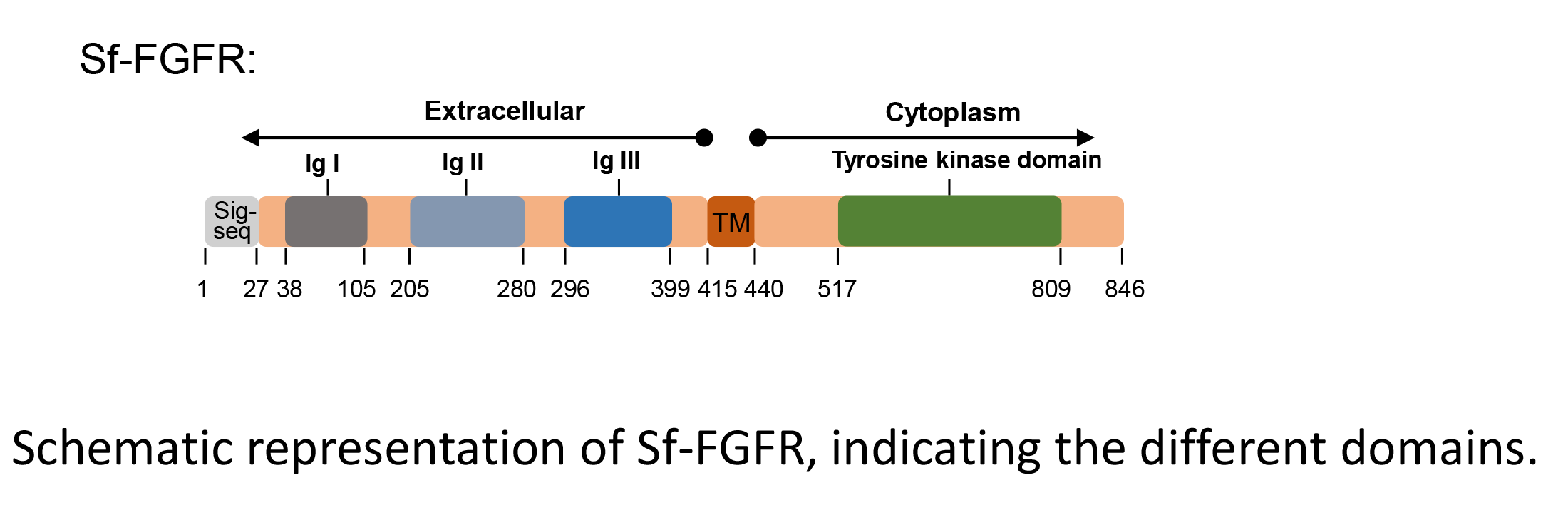

Supplement: Supplementary file 1 [file toxins-10-00546-s001.zip › Supplementary Materials/Figture S2.tif]

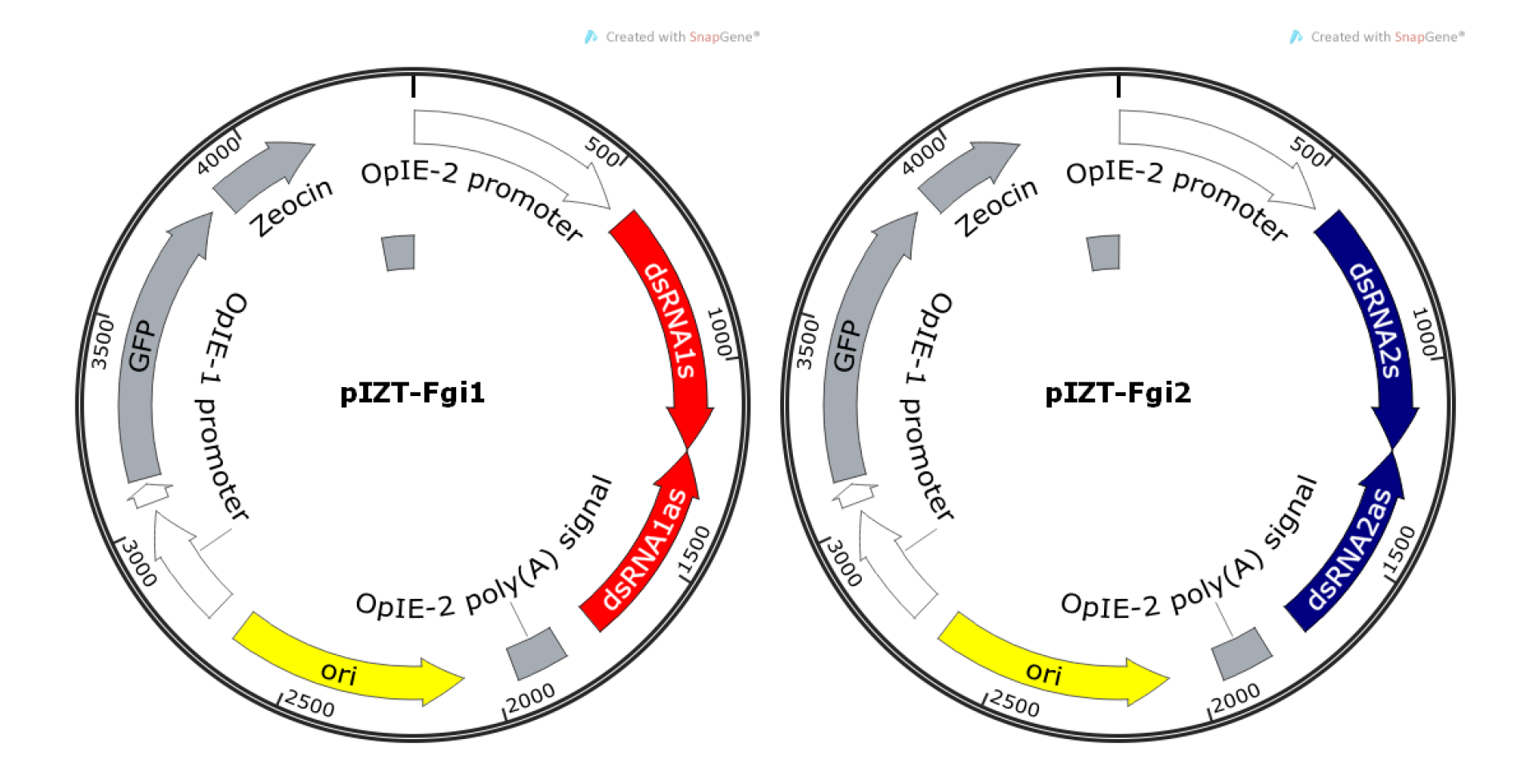

Supplement: Supplementary file 1 [file toxins-10-00546-s001.zip › Supplementary Materials/Figture S3.tif]
